# Co-existence of leiomyomas, adenomyosis and endometriosis in women with endometrial cancer

**DOI:** 10.1038/s41598-020-59916-1

**Published:** 2020-02-27

**Authors:** Sharon E. Johnatty, Colin J. R. Stewart, Deborah Smith, Anthony Nguyen, John O’ Dwyer, Tracy A. O’Mara, Penelope M. Webb, Amanda B. Spurdle

**Affiliations:** 1 Department of Genetics and Computational Biology, QIMR Berghofer Medical Research Institute, Brisbane, Queensland Australia; 2 Department of Histopathology, King Edward Memorial Hospital, Perth, WA Australia; 3 School for Women’s and Infants’ Health, University of Western Australia, Perth, WA Australia; 4 Department of Pathology, The Mater Hospital, Brisbane, Queensland Australia; 5 The Australian e-Health Research Centre, CSIRO, Brisbane, Queensland Australia; 6 Department of Population Health, QIMR Berghofer Medical Research Institute, Brisbane, Queensland Australia

**Keywords:** Cancer, Cancer

## Abstract

Leiomyomas, adenomyosis, and endometriosis are reported to be risk factors for endometrial carcinoma (EC), and adenomyosis and endometriosis also for ovarian carcinoma (OC). We aimed to describe the prevalence of these conditions in EC patients with or without an OC diagnosis, and to investigate their relationship with EC risk and prognostic factors in these patients. We evaluated the co-existence of these three conditions in 1399 EC patients, and compared the prevalence of epidemiological risk factors and tumor prognostic features in patients with each condition versus not. Prevalence of conditions was also assessed in the subset of patients with prior/concurrent OC. The observed coexistence of leiomyomas, adenomyosis and endometriosis significantly deviated from that expected (P = 1.2 × 10^−8^). Patients were more likely to: report a younger age at menarche (P_Trend_ = 0.004) if they had leiomyomas; have used oral contraceptives (P = 6.6 × 10^−5^) or had ≥2 full-term pregnancies (P_Trend_ = 2.0 × 10^−9^) if they had adenomyosis; be diagnosed with EC at younger age (P = 5.0 × 10^−11^) if they had endometriosis. Patients with prior/concurrent OC were more likely to be diagnosed at younger age (P = 5.0 × 10^−5^), have endometriosis (P = 9.9 × 10^−7^), and present with higher stage EC (P_Trend_ = 6.6 × 10^−5^). These findings justify further consideration of these gynecologic conditions as independent risk and prognostic factors for EC.

## Introduction

Endometrial carcinoma (EC) has a worldwide incidence of 8.4 per 100,000 persons per year, and is a leading cause of cancer mortality in women in developed countries^1,2^. Incidence rates of EC are on the rise in developing countries due to increasing levels of obesity and aging populations^3^. Epidemiologic risk factors for EC include age, exogenous estrogen use, and hyperestrogenic status due to obesity, early menarche age, nulliparity, and late-onset menopause^4^. Gynecological conditions reported to increase the risk of EC include polycystic ovary syndrome and uterine leiomyomas (also termed fibroids). Endometriosis was also shown to be robustly associated with up to a 3-fold increased risk of clear cell and endometrioid ovarian carcinoma (OC) in large-scale meta-analysis of self-reported data^5^. However, evidence to support endometriosis as a risk factor for EC from individual studies is conflicting;^6–12^ risk estimates ranged from 0.78 to 2.8, and interpretation of findings has been complicated by differential consideration of endometriosis coincidental to EC diagnosis, and also by differing adjustment for confounders.

Adenomyosis is a benign gynecological condition described as an extension of endometrial tissue (glands and stroma) into the myometrium. There is evidence that adenomyosis is correlated with uterine leiomyomas^13^. The prevalence of adenomyosis at hysterectomy has been reported to be 20% to 30%^14^. An analysis of hysterectomy samples identified adenomyosis in 66% of 197 patients found to have EC^15^, and a recent review identified 78 case reports that describe EC arising from adenomyotic foci^16^. Evidence for an association of adenomyosis with both OC and EC, irrespective of endometriosis, has been provided by unadjusted analyses of medical records from the National Insurance Research database of Taiwan^17,18^. It is unclear if tumor prognostic features or survival differ for EC associated with concurrent adenomyosis^19,20^.

We noted that the presence of uterine leiomyomas, adenomyosis and endometriosis may be correlated, and the overlap in reported epidemiological risk factors for these conditions and for EC. To date no study has dissected the correlation between all three conditions, and their individual and combined relationship with epidemiological risk factors for EC. We conducted a case-only descriptive analysis that: (i) assessed co-existence of leiomyomas, adenomyosis and endometriosis in EC patients; (ii) compared EC risk factors and tumor prognostic features for women with EC identified to have no evidence of leiomyomas, adenomyosis or endometriosis versus subgroups annotated as having one, two or all three of these conditions at/before EC diagnosis; (iii) evaluated overall and EC-specific survival for these patient subgroups; (iv) assessed if these three conditions were overrepresented in the subset of women with report of OC prior to or concurrent with their EC diagnosis.

## Results

### Comparison of self-reported and pathology-detected conditions

A total of 298 patients self-reported leiomyomas (presumed symptomatic, 216 confirmed in pathology reports), and an additional 519 patients had leiomyomas in their pathology reports (presumed asymptomatic or undiagnosed prior to EC) (Fig. S1). For endometriosis, 79 patients self-reported having endometriosis (18 had endometriosis in the ovaries from pathology reports), and an additional 100 patients were identified only from information contained in hysterectomy pathology reports (Fig. S2). There was adenomyosis in a total of 572 (41.0%) EC patients based on pathology reports only (Fig. S3).

The prevalence of risk factors was not significantly different for patients with self-reported conditions versus those where the conditions were identified only from pathology reports (Table S2), with the exception of age at EC diagnosis. Women with EC who self-reported leiomyomas were significantly younger at their EC diagnosis compared to those with leiomyomas identified only in pathology reports (mean age at EC diagnosis 60.2 years vs 62.5 years; P = 0.0005). All subsequent analyses of endometriosis and leiomyomas were therefore based on a combined dataset, 817 (58.4%) patients with leiomyomas and 179 (12.8%) patients with endometriosis, adjusting for age at EC diagnosis.

### Co-existence of the three conditions

**Figure 1:**
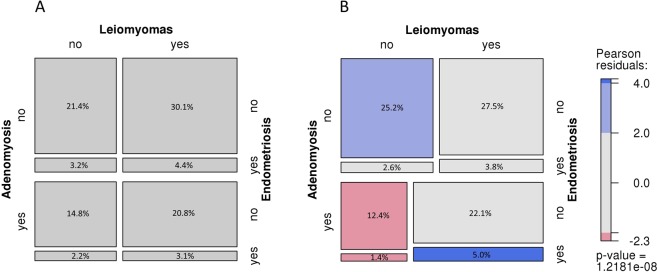
Mosaic plot of co-occurrences of leiomyomas, adenomyosis and endometriosis. Mosaic plot of expected (**A**) and observed (**B**) frequencies of comorbidities of leiomyomas, adenomyosis and endometriosis in EC patients (n = 1399). Tiles sizes are proportional to the (**A**) expected and (**B**) observed frequencies of 3-way subsets of conditions among all EC patients (n = 1399). In panel B, shading indicates Pearson residuals from chi-square test for independence of gynecological conditions; blue indicates positive residuals and pink indicates negative residuals; colour intensity correlates with the size of the residuals; overall p-value suggests strong evidence for departure from the null.

The number and proportion of individuals with none, one, two or all three of the conditions are shown in Table S3, and the deviation of observed versus expected proportions are illustrated as a Mosaic plot in Fig. 1. There was a strong correlation between the conditions (overall P = 1.2 × 10^−8^), driven by presence or absence of all three conditions (blue tiles). There were significantly fewer reports of adenomyosis in women without leiomyomas (pink tiles); this negative correlation did not differ by presence of endometriosis. Considering conditions in pairs (Table S4), just under half (46.4%, 379/817) of patients with leiomyomas also had adenomyosis, and those with adenomyosis were more likely to have leiomyomas than those without adenomyosis (66.3% vs 53.0%, P = 7.0 × 10^−7^). Similarly, half the patients with endometriosis (49.7%, 89/179) were found to have adenomyosis, and those with adenomyosis were more likely to have endometriosis compared to those without adenomyosis (15.6% vs 10.9%). Patients with leiomyomas more often had endometriosis than those without leiomyomas (15.1% vs 9.8%). Secondary analysis restricting to self-reported conditions only (Table S4) showed direction and evidence for correlations remained, albeit reaching different levels of significance.

### Association of each condition with endometrial cancer epidemiologic risk factors and tumor prognostic features

We conducted exploratory analysis to assess if correlation of an EC risk factor with a condition might underlie previously reported associations between that condition and EC risk. Table 1 shows comparisons of risk factors for EC patients with versus without leiomyomas, adenomyosis or endometriosis, adjusted for age at EC and the remaining two conditions. Compared to those without leiomyomas, women with leiomyomas tended to report a younger age at menarche, and were also somewhat more likely to be nulliparous (19% vs 15%). Compared to EC patients without adenomyosis, patients with adenomyosis were significantly more likely to have used oral contraceptives (74% vs 63%, P = 6.6 × 10^−5^), or to have had ≥2 full-term pregnancies (79% vs 68%; P_Trend_ = 1.9 × 10^−9^). EC patients with endometriosis were significantly more likely to be diagnosed with EC at a younger age than those without endometriosis (mean age at EC diagnosis 57 years vs 62 years; P = 5.0 × 10^−11^). While women with endometriosis were more likely than those without endometriosis to be nulliparous (23.5% vs 16.8%) or uniparous (15.6% vs 9.2%), this association was confounded by age at EC diagnosis and presence of the remaining two conditions. There was some evidence that EC patients with endometriosis were less likely to have BMI ≥30 compared to those without endometriosis (44.7% vs 50.3%). All other characteristics were similar between groups. Applying the *a priori* Bonferroni threshold set at P < 0.0008, only the associations between adenomyosis and oral contraceptive use, ≥2 full-term pregnancies, and age remained significantly associated with EC risk.

**Table 1:** Comparison of epidemiological characteristics between endometrial carcinoma patients with or without leiomyomas, adenomyosis or endometriosis.

Characteristic	EC patients without Leiomyomas (n = 582)	EC patients with Leiomyomas (n = 817)	^b^P	EC patients without adenomyosis (n = 827)	EC patients with adenomyosis (n = 572)	^b^P	EC Patients without endometriosis (n = 1220)	EC patients with endometriosis (n = 179)	^b^P
	^a^N (%)	^a^N (%)		^a^N (%)	^a^N. (%)		^a^N (%)	^a^N (%)	
**Mean age at EC diagnosis (range)**	60.7 (26.4–80.0)	61.6 (28.9–80.0)	0.02	61.6 (26.4–80.0)	60.8 (31.9–80.0)	0.2	61.9 (26.4–80.0)	57.0 (28.9–78.9)	5.0 × 10^−11^
**Body Mass Index**									
<25	138 (23.8)	209 (25.6)		211 (25.7)	136 (23.8)		292 (24.1)	55 (30.7)	
25–29.9	153 (26.4)	202 (24.8)		225 (27.4)	130 (22.8)		311 (25.6)	44 (24.6)	
≥30	289 (49.8)	401 (49.2)	0.6	385 (46.9)	305 (53.4)	0.05	610 (50.3)	80 (44.7)	0.03
**Oral Contraceptive use**									
never	198 (34.0)	258 (31.6)		307 (37.1)	149 (26.1)		402 (33.0)	54 (30.2)	
ever	384 (66.0)	558 (68.4)	0.4	520 (62.9)	422 (73.9)	6.6 × 10^−5^	817 (67.0)	125 (69.8)	0.3
**Parity**									
0	89 (15.3)	158 (19.3)		182 (22.0)	65 (11.4)		205 (16.8)	42 (23.5)	
1	64 (11.0)	76 (9.3)		86 (10.4)	54 (9.4)		112 (9.2)	28 (15.6)	
≥2	429 (73.7)	583 (71.4)	0.01	559 (67.6)	453 (79.2)	1.9 × 10^−9^	903 (74.0)	109 (60.9)	0.07
**Age at Menarche**									
≤11	108 (18.8)	206 (25.4)		180 (21.9)	134 (23.7)		272 (22.5)	42 (23.5)	
12–13	284 (49.4)	382 (47.1)		398 (48.5)	268 (47.4)		577 (47.8)	89 (49.7)	
≥14	183 (31.8)	223 (27.5)	0.004	243 (29.6)	163 (28.8)	0.9	358 (29.7)	48 (26.8)	1
**Smoking**									
never	369 (63.5)	539 (66.0)		523 (63.3)	385 (67.3)		792 (65.0)	116 (64.8)	
ever	212 (36.5)	278 (34.0)	0.6	303 (36.7)	187 (32.7)	0.1	427 (35.0)	63 (35.2)	0.7
^**c**^**Family History any cancer (FDR &/or SDR)**									
no	213 (36.6)	285 (34.9)		295 (35.7)	203 (35.5)		445 (36.5)	53 (29.6)	
yes	369 (63.4)	532 (65.1)	0.6	532 (64.3)	369 (64.5)	0.9	775 (63.5)	126 (70.4)	0.06

EC, endometrial carcinoma; FDR, first-degree relative, SDR, second-degree relative.

^a^Ns may not sum to the total because of missing or unknown data; proportions (%) sum to 100% of observations with data available and excludes missing/unknowns.

^b^P-values are from logistic models comparing presence of a condition versus not; models are adjusted for age at EC diagnosis and presence of the other two gynecologic conditions; p-values for BMI, Parity and age at menarche represent trend across ordered groups.

^c^Family history of cancer reported in at least one FDR or SDR.

For transparency, Table S5 shows breakdown of epidemiological and family history risk factors for EC patients with one, two or all three of these conditions (leiomyomas, adenomyosis, endometriosis) versus those with none of these conditions. The overall observations reported above remained unchanged.

Table 2 shows tumor prognostic features for women according to the presence of the three conditions. Recognising that sample sizes for some subgroups are too small to provide reliable estimates, the proportion of grade 1–2 EC was somewhat lower for women with no conditions (68.5%) vs any one of the conditions (73.6% to 89.5%). There was no convincing evidence that non-endometrioid tumors were enriched in patients with these conditions e.g. non-endometrioid tumors comprised 17.3% of the subtypes presenting for individuals with no conditions, with the proportion in other subgroups ranging from 5.3% (adenomyosis and endometriosis) to 20.8% (leiomyomas and endometriosis).

**Table 2:** Comparison of tumor characteristics between endometrial carcinoma patients with none versus any combination of leiomyomas, adenomyosis or endometriosis.

Tumor Characteristic	None	Leiomyomas only	Adenomyosis only	Endometriosis only	Leiomyomas & Adenomyosis	Leiomyomas & Endometriosis	Adenomyosis & Endometriosis	All three conditions
	^a^N (%)	^a^N (%)	^a^N (%)	^a^N (%)	^a^N (%)	^a^N (%)	^a^N (%)	^a^N (%)
Subset of all cases (n = 1399)	352 (25.2)	385 (27.5)	174 (12.4)	37 (2.6)	309 (22.1)	53 (3.8)	19 (1.4)	70 (5.0)
**Tumor Histology & Grade**								
Endometrioid Grade 1	152 (43.2)	185 (48.1)	95 (54.6)	18 (48.6)	191 (61.8)	25 (47.2)	12 (63.2)	47 (67.1)
Endometrioid Grade 2	89 (25.3)	100 (26.0)	51 (29.3)	10 (27.0)	67 (21.7)	14 (26.4)	5 (26.3)	13 (18.6)
Endometrioid Grade 3	50 (14.2)	35 (9.1)	7 (4.0)	4 (10.8)	18 (5.8)	3 (5.7)	1 (5.3)	2 (2.9)
Serous (>5%)	33 (9.4)	32 (8.3)	11 (6.3)	4 (10.8)	19 (6.1)	7 (13.2)	1 (5.3)	4 (5.7)
Clear Cell (>10%), no serous	10 (2.8)	14 (3.6)	5 (2.9)	0 (0.0)	4 (1.3)	2 (3.8)	0 (0.0)	0 (0.0)
Carcinosarcoma (MMMT)	13 (3.7)	18 (4.7)	2 (1.1)	0 (0.0)	7 (2.3)	0 (0.0)	0 (0.0)	4 (5.7)
^b^Other epithelial	5 (1.4)	1 (0.3)	3 (1.7)	1 (2.7)	3 (1.0)	2 (3.8)	0 (0.0)	0 (0.0)
**FIGO stage**								
I	263 (75.8)	311 (81.0)	149 (85.6)	29 (78.4)	282 (91.3)	42 (80.8)	18 (94.7)	62 (88.6)
II	32 (9.2)	31 (8.1)	15 (8.6)	1 (2.7)	9 (2.9)	4 (7.7)	1 (5.3)	5 (7.1)
III	39 (11.2)	35 (9.1)	10 (5.7)	6 (16.2)	17 (5.5)	5 (9.6)	0 (0.0)	2 (2.9)
IV	13 (3.7)	7 (1.8)	0 (0.0)	1 (2.7)	1 (0.3)	1 (1.9)	0 (0.0)	1 (1.4)
**Lymphovascular Space Involvement**								
No/Unknown	259 (73.6)	298 (75.1)	148 (85.1)	27 (73.0)	261 (84.5)	41 (77.4)	17 (89.5)	59 (84.3)
Yes	93 (26.4)	96 (24.9)	26 (14.9)	10 (27.0)	48 (15.5)	12 (22.6)	2 (10.5)	11 (15.7)

MMMT, malignant mixed Müllerian tumor; FIGO, International Federation of Gynecology and Obstetrics.

^a^Ns may not sum to the total because of missing or unknown data; proportions (%) sum to 100% of observations where data was available and excludes missing/unknowns.

^b^Other epithelial include mixed subtypes where serous or clear cell component does not reach the percentage noted, or where the histology was unknown (2 individuals were diagnosed by curettage).

### Prevalence of conditions in EC patients with prior or concurrent OC diagnosis

We identified a subset of 30 EC patients who also had an OC diagnosis; 28 had OC concurrent with EC, and two were diagnosed prior to EC. Table S6 summarizes ovarian tumor characteristics and coexisting gynecologic conditions of these EC-OC patients. Table 3 shows comparisons of the characteristics of EC patients with OC versus those without OC, including: evidence for the three gynecological conditions under study, epidemiological risk factors, tumor characteristics, and family cancer history. There were no differences between patients with or without OC for presence of leiomyomas or adenomyosis, irrespective of other conditions. A significantly greater proportion of EC patients with concurrent/prior OC had endometriosis compared to those without OC (43.3% vs 12.1%; P = 2.1 × 10^−4^). Considering the conditions in combination (data not shown), proportions were significantly different between patients with prior/concurrent OC versus those without OC for endometriosis only (16.7% vs 2.3%, P = 9.9 × 10^−7^) and for co-existence of all three conditions (20.0% vs 4.7%; P = 0.0001).

**Table 3:** Characteristics of EC patients with and without prior/concurrent ovarian cancer.

Characteristics	EC patients - no Ovarian Cancer (N = 1369)	^a^EC patients with Ovarian Cancer (N = 30)	^c^P
	^b^N (%)	^b^N (%)	
**Endometriosis**	166 (12.1)	13 (43.3)	2.1 × 10^−4^
**Leiomyomas**	799 (58.4)	18 (60.0)	0.6
**Adenomyosis**	561 (41.0)	11 (36.7)	0.7
**Mean age at EC diagnosis (range)**	61.5 (26.4–80.0)	54.4 (33.3–75.9)	5.0 × 10^−5^
**Body Mass Index**			
<25	340 (25.0)	7 (23.3)	
25–29.9	346 (25.4)	9 (30.0)	
≥30	676 (49.6)	14 (46.7)	0.8
**Oral Contraceptive use**			
never	448 (32.7)	8 (26.7)	
ever	920 (67.3)	22 (73.3)	0.9
**Parity**			
0	237 (17.3)	10 (33.3)	
1	132 (9.6)	8 (26.7)	
≥2	1,000 (73.0)	12 (40.0)	0.03
**Age at Menarche**			
≤11	305 (22.5)	9 (30.0)	
12–13	651 (48.0)	15 (50.0)	
≥14	400 (29.5)	6 (20.0)	0.3
**Smoking**			
never	890 (65.1)	18 (60.0)	
ever	478 (34.9)	12 (40.0)	0.8
^**d**^**Family History of any cancer**			
no	492 (35.9)	6 (20.0)	
yes	877 (64.1)	24 (80.0)	0.07
^**d**^**Family History of breast cancer**			
no	961 (70.3)	14 (46.7)	
yes	406 (29.7)	16 (53.3)	0.003
^**d**^**Family History of any Lynch cancer**			
no	665 (48.6)	13 (43.3)	
yes	704 (51.4)	17 (56.7)	0.7
^**e**^**Tumor Histology & Grade**			
Endometrioid Grade 1	712 (52.0)	13 (43.3)	0.3
Endometrioid Grade 2	339 (24.8)	10 (33.3)	0.3
Endometrioid Grade 3	119 (8.7)	1 (3.3)	0.3
Serous (>5%)	110 (8.0)	1 (3.3)	0.3
Clear Cell (>10%), no serous	34 (2.5)	1 (3.3)	0.8
Carcinosarcoma	41 (3.0)	3 (10.0)	0.03
Other epithelial	14 (1.0)	1 (3.3)	0.2
**FIGO stage**			
I	1,140 (83.7)	16 (53.3)	
II	93 (6.8)	5 (16.7)	
III	107 (7.9)	7 (23.3)	
IV	22 (1.6)	2 (6.7)	6.6 × 10^−5^
**Lymphovascular Space Involvement**			
No/Unknown	1081 (79.0)	20 (66.7)	
Yes	288 (21.0)	10 (33.3)	0.07

EC, endometrial cancer.

^a^Ovarian cancer was concurrent with EC in 28 patients and prior to EC in 2 patients; Tumor mismatch repair (MMR) status was known for 17 individuals;12 were MMR proficient, 4 were MMR deficient due to MLH1 somatic inactivation (methylation, somatic variant), and 1 MMR deficient individual carried an MLH1 truncating variant considered pathogenic.

^b^Ns may not sum to the total because of missing or unknown data; proportions (%) sum to 100% of observations where data available and excludes missing/unknowns. Ns for endometriosis, leiomyomas and adenomyosis are for each individual condition irrespective of coexistence of either of the other two

^c^P-values are P-Trend across ordered groups (BMI, Parity, age at menarche, FIGO stage) or logistic regression models for binary variables adjusted for age at EC diagnosis; P-values for tumor histology subtypes are from comparison of proportions.

^d^Cancer reported in at least one first- degree or second-degree relative; Lynch cancers are cancer of the bile duct, bladder, brain, colon/rectum, duodenum, endometrium, gastrointestinal/GI, ovary/fallopian tube, pancreas, renal pelvis, stomach.

^e^Endometrioid tumor histology and grade were combined into a single variable for Endometrioid Grades 1, 2, or 3; other epithelial include mixed subtypes where serous or clear cell component does not reach % noted, or where the histology was unknown (2 individuals diagnosed by curettage).

EC patients with OC were significantly more likely to be diagnosed with EC at a younger age compared to those without OC (mean EC diagnosis age 54.4 vs 61.5; P = 5.0 × 10^−5^). They were also more likely to be nulliparous (33% vs 17%) or uniparous (26.7% vs 9.6%) compared to those without OC. Family history of cancer was somewhat higher among EC patients with OC versus those without, accounted for by the difference in family history of breast cancer specifically (53.3% vs 29.7%). Women with prior or concurrent OC were significantly more likely to present with higher stage EC (P_Trend_ = 6.6 × 10^−5^). There was also evidence that this subgroup of patients were more likely to present with poor prognosis carcinosarcoma (10.0% vs 3.0% in the reference group), a relationship independent of stage.

Considering the subset of 13 EC-OC patients with endometriosis (43%), a single patient reported prior ovarian malignancy (a granulosa cell tumor diagnosed 2 years prior to EC presenting as a carcinosarcoma). The remaining 12 patients with concurrent EC-OC diagnoses presented with EC histological subtype endometrioid (n = 10), carcinosarcoma (n = 1), and mixed endometrioid-other (n = 1); OC histological subtype was endometrioid or mixed including an endometrioid component for all 12 patients, and endometriosis within the ovary was noted in 10 of these women (Table S6).

### Association of conditions with EC-specific and overall survival

We also evaluated EC-specific and overall survival for each condition individually, and in combination with other conditions (Table 4). Presence of leiomyomas was marginally associated with better EC-specific (HR 0.67; 95% CI 0.47–0.96) and overall (HR 0.68; 95% CI 0.51–0.91) survival after adjustment for known prognostic indicators. This observation was carried through when considering the conditions in combination, with improvement in survival observed for women with leiomyomas only (EC-specific HR 0.58 (0.37–0.89); overall survival HR 0.57; 95% CI 0.39–0.83). There was no difference in survival for the small subgroup of women with prior or concurrent OC.

**Table 4:** Overall and EC-specific survival in patients according to gynecologic conditions.

EC Patient subgroup	N Died (%)	EC-specific Survival		Overall Survival	
		^a^HR (95% CI)	P	^a^HR (95% CI)	P
^**b**^**Individual conditions**					
Leiomyomas (n = 817)	94 (11.5)	0.67 (0.47–0.96)	0.027	0.68 (0.51–0.91)	0.011
Adenomyosis (n = 572)	56 (9.8)	0.67 (0.44–1.04)	0.074	0.88 (0.63–1.21)	0.431
Endometriosis (n = 179)	23 (12.8)	0.74 (0.39–1.40)	0.354	1.19 (0.75–1.88)	0.458
**Coexisting conditions**					
None (n = 352)	71(20.2)	1.00		1.00	
Leiomyomas only (n = 385)	50 (13.0)	0.58 (0.37–0.89)	0.012	0.57 (0.39–0.83)	0.003
Adenomyosis only (n = 174)	20 (11.5)	0.52 (0.25–1.07)	0.076	0.77 (0.46–1.28)	0.3
Endometriosis only (n = 37)	4 (10.8)	0.47 (0.14–1.54)	0.21	0.56 (0.20–1.55)	0.3
Leiomyomas & Adenomyosis (n = 309)	27 (8.7)	0.55 (0.31–0.98)	0.043	0.60 (0.38–0.95)	0.03
Leiomyomas & Endometriosis (n = 53)	10 (18.9)	0.79 (0.31–2.00)	0.616	1.21 (0.62–2.38)	0.6
Adenomyosis & Endometriosis (n = 19)	2 (10.5)	1.03 (0.14–7.54)	0.978	1.28 (0.31–5.27)	0.7
All three conditions (n = 70)	7 (10.0)	0.25 (0.06–1.08)	0.064	0.74 (0.33–1.66)	0.5
**Prior/Concurrent Ovarian Cancer**					
No ovarian cancer (n = 1369)	184 (13.4)	1.00		1.00	
Prior/Concurrent ovarian cancer (n = 30)	7 (23.3)	0.55 (0.16–1.90)	0.343	1.18 (0.52–2.68)	0.7

EC, endometrial carcinoma; HR, Hazard Ratio; CI, confidence Interval; LVSI, lymphovascular space involvement.

^a^Estimates are based on Cox modelling of factor variables using left-truncation; covariates includes age at EC diagnosis (continuous), FIGO stage I, II, III + IV (factor variables); histology endometrioid Grade 1, endometrioid Grade 2, endometrioid Grade 3, serous, clear cell, MMMT, other epithelial (factor variables) and LVSI no/unknown, yes.

^b^Analysis of EC patients with specified condition vs not, irrespective of presence of another condition.

## Discussion

Our descriptive analysis of leiomyomas, adenomyosis and endometriosis in 1399 EC patients participating in the ANECS population-based study highlights how common these conditions are in EC patients, provides evidence for their co-occurrence, and describes their relationship with known EC risk and prognostic factors. Given the known association between endometriosis and OC, we also present a secondary exploratory analysis of these conditions in a subset of EC patients with prior or concurrent OC.

We found evidence for leiomyomas in 58.4% of our EC patients, of which a large proportion (63.5%) were identified from pathology reports only and presumed to be asymptomatic. The latter observation may explain the extremely wide ranges previously reported for prevalence of this condition — from 5% to 77% across studies of different study designs, populations and diagnostic techniques^21^. The observation that women with leiomyomas have earlier age at menarche and increased nulliparity suggests these two factors may underlie, at least in part, documented associations between self-reported leiomyomas and increased EC risk.

We identified adenomyosis in 41% of EC patients, compared to 20–30% observed in women without EC undergoing hysterectomy^14^. This is consistent with another study reporting increased prevalence of adenomyosis in the small subset of women identified to have EC at hysterectomy. Also consistent with previous reports^13,14^, EC patients with adenomyosis were more likely to be multiparous — a factor associated with *decreased* EC risk^22^. We also observed that EC patients with adenomyosis were significantly more likely to have used oral contraceptives, a relationship that may reflect the fact that oral contraceptives may be prescribed for treatment of adenomyosis^23^. However, oral contraceptive use is associated with *reduced risk* of EC^4^. Thus, previous reports of an association between adenomyosis and increased risk of EC are unlikely to be explained by associations between adenomyosis and known epidemiological risk factors for EC.

Our observation that 12.8% of EC patients had endometriosis, including presumed asymptomatic disease detected at surgical pathology review (55.9% of this subset), is broadly similar to previous reports of 10% in the general population, and less than reported figures of 30% to 87% in women presenting with chronic pelvic pain^24^. Although unadjusted analyses indicated that women with endometriosis are more likely to be nulliparous and less likely to have ≥2 livebirths, the association was not significant after adjustment for age. This suggests that effects of endometriosis on fertility are not likely to mediate any association between endometriosis and EC.

Regarding co-existence of conditions, we provide convincing evidence that the three conditions are correlated (Fig. 1, Tables S3 and S4). It may be argued that this correlation is influenced by the reporting practice of individual pathologists in terms of review and diagnosis of concurrent benign conditions in EC patients. However, the overall correlations remained for analysis of self-reported leiomyomas, self-reported endometriosis and pathology-extracted adenomyosis (Table S4), suggesting that diagnostic bias does not entirely account for these findings. Overall, the current findings indicate that it will be important for future case-control studies to investigate all three conditions simultaneously, to delineate which specific features may be predictors of EC risk.

While the tumor prognostic features of women did not differ markedly depending on whether they had one or more conditions, multivariate analysis adjusting for known prognostic indicators provides some evidence that presence of leiomyomas is associated with improved survival after EC diagnosis. Further studies to validate this finding, and to provide biological rationale for this association, will be important to determine if recognition of leiomyomas before or at surgery may be considered as an additional independent prognostic indicator when considering surgical and chemotherapeutic interventions for EC patients.

Regarding the relationship of conditions with concurrent or prior OC, although EC patients with OC were more likely to present with all three conditions compared to women without OC (20% vs 4.7%), but the overwhelming difference between the two groups was for endometriosis (Table 3). This observation suggests that, of the three conditions, endometriosis is most worthy of further study in relation to development of EC after or concurrent with an OC diagnosis. While it could be argued that endometriosis-related infertility could mediate risk associations between endometriosis and EC (and also OC), we showed that women with endometriosis were not more likely to be nulliparous after adjustment for age.

Detailed review of the characteristics of each of the 30 EC patients with prior or concurrent OC (Table S6) revealed that 13 (43%) women had endometriosis, with OC histological subtype being endometrioid or mixed histologies that included clear cell and endometrioid carcinoma components. This observation supports previous reports that endometriosis is a risk factor for ovarian tumors of endometrioid and clear cell subtype^8,25,26^.

Our case-only study was not able to directly estimate EC risk associated with leiomyomas, adenomyosis and endometriosis. Estimates of EC risk and prognosis associated with benign gynaecological conditions would require access to pathology data from women who are unaffected by EC and with an intact uterus, which would preclude them from eligibility as controls in such studies. In particular A prevailing weakness of case-control studies of benign gynecologic conditions is the unlikely scenario where such uterine features are detected through pathology assessment without some indication for hysterectomy or endometrial biopsy. Nevertheless, our findings provide justification for further consideration of these three conditions in the aetiology of EC. EC risk factor prevalence observed in EC subgroups defined by presence of one or more conditions indicates that shared risk factors for EC and these three conditions are unlikely to account totally for previously reported associations of EC (and probably OC) with these three conditions. This is particularly true for adenomyosis; the two factors with significantly increased prevalence in women with pathology-detected adenomyosis are known to be associated with decreased risk of EC. Our analyses were based on patient features derived from a combination of self-reported and pathology reported data, and we acknowledge the likelihood of inaccuracy in self-reported data. We had no self-reported data for adenomyosis, which was obtained entirely from pathology reports. There are no well-established guidelines in relation to the diagnostic criteria for adenomyosis in Australia, and it is likely that different pathologists use varied and possibly subjective criteria for adenomyosis. It is also likely that milder forms of adenomyosis may not have been reported, but moderate or severe adenomyosis should be well recognized^27^. Additionally there may be some variation also in pathology data generated by multiple cancer centres that contributed data to this study without central histology review. However we suggest that such variations would be randomly distributed between comparison groups given the case-only nature of our analysis. Further, random error is likely to bias associations towards the null. We also acknowledge a potential for detection bias where pathology reporting and the detection of EC was incidental to hysterectomy for apparently benign conditions. Previous reports suggest that while this is possible, only 0.2% of endometrial tumors are discovered among hysterectomies performed for benign conditions^28,29^. Indeed this is in accord with anecdotal experiences of collaborating pathologists involved in the ANECS study, and we suggest that it is unlikely that this was a factor in our analysis. Our recommendation is that all three conditions should be investigated more thoroughly as independent risk factors for EC in an appropriately designed study.

Previous studies using a variety of statistical approaches have reported evidence for shared genetic risks between endometriosis and OC^30,31^, and endometriosis and EC^32^. A GWAS of leiomyomas reported shared signals with endometriosis, and also evidence for a shared signal with EC^33^. It is now feasible to consider much larger studies assessing similarity of genetic architecture (genetic correlation) between these conditions and EC and OC, and cross-disease GWAS to investigate evidence of pleiotropy. Further, Mendelian Randomization studies could assess if genetic predictors of the gynecological conditions are also associated with increased gynecological cancer risk, while also addressing shared epidemiological risk factors by using genetic markers of these other risk factors to adjust the analysis. We thus envisage that genetic studies building on GWAS of these conditions are the next obvious step to (re)investigate the role of these conditions in the aetiology of EC and OC.

## Methods

### Study population

Participants included in this case-only analysis were endometrial cancer patients drawn from the Australian National Endometrial Cancer Study (ANECS; see Supplementary Methods for details on study design and data collection). Self-report for leiomyomas and endometriosis was collected by telephone interview. Information on EC pathological features at hysterectomy, including presence of leiomyomas, adenomyosis and endometriosis, were abstracted from diagnostic pathology reports. In addition, we used a natural language processing (NLP) tool to search scanned EC pathology reports for evidence of leiomyomas, adenomyosis and endometriosis (See Supplementary Methods, Table S1 for further details on NLP methods and search terms). Women were not questioned regarding diagnosis of adenomyosis, and such data was extracted from pathology reports only using abstraction and NLP methods.

### Statistical analysis

Using the χ^2^ test for comparison of means and proportions, we compared the prevalence of epidemiologic characteristics of women who self-reported leiomyomas or endometriosis (presumed symptomatic), versus those identified to have leiomyomas or endometriosis from pathology reports only (presumed asymptomatic). The purpose was to investigate if there was any evidence for a different underlying etiology of disease and/or differences in behaviour patterns based on prior knowledge of this condition. Based on findings (see Results), patients were considered to have leiomyomas or endometriosis if they self-reported and/or had pathology confirmation of these conditions.

We documented the proportions of EC patients with co-existing leiomyomas, adenomyosis and/or endometriosis versus those with none of these conditions in a combined dataset (self-reported and pathology reported), in comparison to what might be expected based on the relative proportions of each condition alone for the combined dataset. A mosaic plot illustrating the observed versus expected proportions of women with co-existing leiomyomas, adenomyosis and endometriosis among EC patients was generated in the Visualizing Categorical Data (vcd) package (version 1.4–4)^34^ in the R project for Statistical Computing (http://www.r-project.org/). Secondary analysis was undertaken to assess evidence for correlation between adenomyosis and self-reported data only for the other two conditions.

We compared epidemiological risk factors among EC patients with any one condition (leiomyomas, adenomyosis, or endometriosis) versus those who did not have that condition. Factors compared included mean age at EC diagnosis, body mass index (BMI; <25, 25–29, ≥30 vs kg/m^2^), oral contraceptive use (ever vs never), parity (0, 1, ≥2 pregnancies ≥6 months gestation), age at menarche (≤11, 12–13, ≥14), smoking (ever vs never), and reported family history of cancer (any cancer in ≥1 first- or second-degree relative). Tests for comparison of proportions were adjusted for age at EC diagnosis, and additionally for the remaining two conditions investigated. Secondary analysis compared risk factors for women who had any combination of leiomyomas, adenomyosis, and endometriosis versus those who did not, adjusting for age.

We compared tumor pathology features (tumor stage, FIGO I, II, III, IV), histological subtype, lymphovascular space involvement (LVSI) for women who had any combination of leiomyomas, adenomyosis, and endometriosis versus those who did not. We also described the prevalence of the three conditions, reported risk factors for EC, and tumor prognostic features, for a subset of EC patients identified to have OC diagnosed prior to or concurrent with EC, in comparison to EC patients with no prior/concurrent OC diagnosis. Lastly, we explored overall and EC-specific survival among EC patients with coexisting gynecologic conditions to determine whether any condition was associated with prognosis. We also evaluated women who were diagnosed with OC prior to or concurrent with EC. Cox models were minimally adjusted for age at EC diagnosis as a continuous variable; fully adjusted analyses included tumor stage (FIGO stage I, II, III/IV), tumor histology (endometrioid Grade 1, 2, or 3, serous, clear cell, carcinosarcoma/MMMT, other epithelial), LVSI (present/absent), age at EC diagnosis. Survival time was defined as the interval from date of first treatment for EC to date of death from any cause or due to EC, or censored at 31 December 2013.

Differences between patient subgroups were compared using the t-test for comparison of means, χ^2^ test for comparison of proportions, and trends across ordered groups (for BMI, parity and age at menarche) using the nonparametric Cuzick’s test. For result reporting and interpretation, p-values ≤ 0.05 were denoted for descriptive purposes, while estimates and comparisons with p-values < 0.0008 (nominal p-value 0.05/65 tests) in the main results were reported as significant after applying a Bonferroni correction. All tests were two-tailed, and were performed in STATA SE v. 13 (Stata Corp., USA).

### Ethics statement

All ANECS participants provided informed written consent, and ethical approval was obtained from the QIMR Berghofer Medical Research Institute Human Research Ethics Committee, participating hospitals and cancer registries. ANECS data collection and analysis methods were performed in accordance with relevant guidelines and regulations.

## Supplementary information

Supplementary Information.

## Data Availability

In order to comply with all institutional ethics, data cannot be shared without completely anonymizing all patient data included in this report. The authors are happy to share aggregate data presented in the manuscript upon request, and will consider all reasonable requests to the corresponding author within the confines of institutional ethics committee regulations.
