# Supplementary material for: Co-existence of leiomyomas, adenomyosis and endometriosis in women with endometrial cancer
Source: Sci Rep. 2020 Feb 27;10:3621. doi: 10.1038/s41598-020-59916-1 (PMC7046700; doi:10.1038/s41598-020-59916-1)
Supplement: Supplementary file 1 — Supplementary Information. [file 41598_2020_59916_MOESM1_ESM.docx]

**Supplementary Information**

Co-existence of leiomyomas, adenomyosis and endometriosis in women with endometrial cancer.

Sharon E. Johnatty^1^, Colin J.R. Stewart^2,3^, Deborah Smith^4^, Anthony Nguyen^5^, John O’Dwyer^5^, Tracy A. O’Mara^1^, Penelope M. Webb^6^, Amanda B. Spurdle^1^*

**Supplementary Methods:**

**Australian National Endometrial Cancer Study (ANECS) design and data collection.**

All ANECS participants provided informed written consent, and approval was obtained from the QIMR Berghofer Medical Research Institute Human Research Ethics Committee, participating hospitals and cancer registries. Details of participant ascertainment, eligibility criteria, questionnaires and data collection, including assessment of family history, have been previously reported ^1,2^. After exclusions for ineligibility, a final sample of 1399 patients with primary EC completed an interview administered by telephone. Questionnaires included self-report for leiomyomas and endometriosis, and whether endometriosis was clinically or surgically diagnosed. Information on EC pathological features at hysterectomy and the presence of leiomyomas, adenomyosis and endometriosis were abstracted from diagnostic pathology reports for consenting women. Clinicopathological data including histological subtype, grade, tumor stage, and lymphovascular space invasion (LVSI) were also abstracted from medical/pathology records; tumors were re-staged using the International Federation of Gynecology and Obstetrics (FIGO) 2009 criteria. Following convention regarding grading of “non-endometrioid” tumors, and knowledge of prognostic features of tumors of mixed histology ^3^, tumor histology and grade were combined in a single variable with the following categories: endometrioid grade 1, endometrioid grade 2, endometrioid grade 3, serous (≥5%), clear cell (≥10%, and no serous ≥5%), carcinosarcoma (also termed malignant mixed müllerian tumor, MMMT), and other epithelial. Vital status was determined from medical records and using probabilistic record data linkage to the Australian National Death Index. Survival time was calculated from date of primary treatment for EC to date of death (overall, EC‐specific) or censored at 31 December 2013.

**References**

1 Rowlands, I. J. *et al.* Gynecological conditions and the risk of endometrial cancer. *Gynecologic oncology* **123**, 537-541, doi:10.1016/j.ygyno.2011.08.022 (2011).

2 Johnatty, S. E. *et al.* Family history of cancer predicts endometrial cancer risk independently of Lynch Syndrome: Implications for genetic counselling. *Gynecologic oncology* **147**, 381-387, doi:10.1016/j.ygyno.2017.08.011 (2017).

3 Rabban, J. Mixed Epithelial Carcinoma of the Endometrium: Recommendations for Diagnosis from the ISGyP Endometrial Carcinoma Project. March 2017. ( (accessed June 2019)). <<https://handouts.uscap.org/AN2017/2017_CM19_rabba_0601.pdf>>.

**Natural Language Processing tool**

Scanned pathology reports were obtained as electronic portable document format (PDF) images. In order to convert the PDF images into searchable PDF text, Adobe Acrobat Pro was used to apply optical character recognition (OCR) on each report.

A natural language processing (NLP) tool was developed to search within the PDF text to extract evidence of leiomyomas, endometriosis, and adenomyosis (see **Table S1** for a list of inclusion and exclusion search terms). These terms were expanded with their character variations based on spelling variations as well as common OCR errors (e.g., ‘l’ can be ‘i,’ and ‘m’ can also be ‘rn’). The matching of search terms was case-sensitive and based on the longest possible text string match starting from any position.

For each instance of evidence found in the PDF text, the evidence was assigned one of three values (yes, no, not reported) for each of the three gynecological conditions, i.e. if search terms were identified in the pathology report, then the corresponding variable was assigned a ‘yes’ value. Common negated assertion phrases containing the search terms were also extracted (e.g. ‘adenomyosis: absent’). If these phrases were identified in the PDF, then the corresponding variable was assigned a ‘no’ value. If more than one conflicting value was generated by the tool for a given pathology report (i.e. ‘yes’ and ‘no’ values), then the tool would output a ‘conflict’ value, which was then be manually resolved by reviewing the relevant pathology report. However, if no search terms were found or were from the ‘exclusion’ list, then the patient was assigned a ‘not reported’ value. The NLP tool also provided the context or statements surrounding each search term found in the pathology report. The context could be used to assist with quality assurance checks.

The NLP tool’s output was crosschecked against the original manually abstracted coding, and discordances were resolved by review of the pathology report in question. If a term was identified by NLP, but the pathology abstraction code was no/unknown, then the extracted context provided by the NLP tool was reviewed and a decision made as to whether or not the condition was reported by the pathologist. If a term was identified by pathology abstraction but not identified by NLP, then the entire pathology report was re-reviewed and a determination made as to the reasons for this discordance and the correct coding. Discordances between NLP and manual abstraction were predominantly because of poor quality of scanned pathology reports, or markings in the text that prohibited NLP recognition of text characters. ‘Conflict’ values were observed for three patients for NLP of adenomyosis all of whom had the statement ‘evidence of adenomyosis: absent’ as well as further comment by the treating pathologist on adenomyosis of the myometrium. The final decision was based on a comprehensive review of pathology reports. There were no ‘conflict’ values for either endometriosis or leiomyomas.

Overall the NLP tool was observed to have high concordance with abstracted data, with agreements between NLP and abstracted data of 94.6% for adenomyosis, 97.9% for endometriosis, and 75.5% for leiomyomas. The NLP tool and re-review of pathology reports to resolve discordance between NLP and abstraction aided in identifying the presence of conditions missed by human abstraction (41 for adenomyosis, 16 for endometriosis, and 76 for leiomyomas). Further details of the performance of NLP are captured in a parallel manuscript (Nguyen et al, manuscript in preparation).

**Figure S1**. **Flow chart showing selection of EC patients with leiomyomas using patient self-reports and data obtained from NLP tool and review of EC pathology reports**. EC=endometrial cancer; NLP=natural language processing; 298 EC patients self-reported leiomyomas, 216 of which were confirmed in pathology review and 1101 did not (1090 reported no leiomyomas, and 11 reported ‘unknown’). An additional 519 women among those who did not self-report leiomyoma had evidence of leiomyomas in their pathology reports. A total of 817 EC patients (735 confirmed in pathology reports) were considered for leiomyoma analysis.

**Figure S2**. **Flow chart showing selection of EC patients with endometriosis using patient self-reports and data obtained from NLP tool and review of EC pathology reports**. EC=endometrial cancer; NLP=natural language processing; 79 EC patients self-reported endometriosis (16 were self-reported by patients as being clinically diagnosed without histological confirmation, and another 22 as being surgically diagnosed). Of the 79 women self-reporting endometriosis, 18 were confirmed in pathology reports examining tissue resected at hysterectomy. An additional 100 women were identified to have endometriosis from information contained in hysterectomy pathology reports. Of the remaining 1220 EC patients, 1213 self-reported no endometriosis, with pathology reports specifically noting no evidence of endometriosis for 14 of these. A total of 179 EC patients (118 confirmed in pathology reports) were considered for endometriosis analysis.

**Figure S3**. **Flow chart showing selection of EC patients with adenomyosis**. Patients did not self-report on adenomyosis; selection was therefore based on abstracted data confirmed with NLP tool, and additional cases identified with NLP tool and review of EC pathology reports. EC=endometrial cancer; NLP=natural language processing. A total of 572 EC patients were found to have adenomyosis.

| **Table S1: NLP Inclusion and exclusion search terms** | |  |  |
| --- | --- | --- | --- |
|  |  |  |  |
| **Evidence Type** | **Leiomyoma** | **Endometriosis** | **Adenomyosis** |
| **Inclusion search terms** | fibroid | endometriosis | adenomyosis |
|  | fibroids |  | adenomyotic |
|  | leiomyoma |  |  |
|  | leiomyomata |  |  |
|  | leiomyomas |  |  |
|  | smooth muscle neoplasm |  |  |
|  | smooth muscle tumour |  |  |
|  | smooth muscle tumor |  |  |
| **Exclusion search terms** | fibrosis | endometritis |  |
|  | fibrotic |  |  |

Table S2: Characteristics of EC patients according to self-reported (presumed symptomatic) vs pathology reported only (presumed asymptomatic) leiomyomas and endometriosis

| **Characteristic** | **Endometriosis self-reported (N=79)** | | **Endometriosis pathology reported only (N=100)** | | **^b^P** | **Leiomyomas self-reported only (N=298)** | | **Leiomyomas pathology reported only (N=519)** | | **^b^P** |
| --- | --- | --- | --- | --- | --- | --- | --- | --- | --- | --- |
|  | **^a^N** | **(%)** | **^a^N** | **(%)** |  | **^a^N** | **(%)** | **^a^N** | **(%)** |  |
| **Mean age at EC diagnosis (range)** | 55.6 (36.3-76.8) |  | 58.1 (28.9-78.9) |  | 0.09 | 60.2 (32.0-80.0) |  | 62.5 (28.9-80.0) |  | 0.0005 |
| **BMI** |  |  |  |  |  |  |  |  |  |  |
| <25 | 24 | (30.4) | 31 | (31.0) |  | 83 | (27.9) | 126 | (24.5) |  |
| 25-29.9 | 18 | (22.8) | 26 | (26.0) |  | 71 | (23.9) | 132 | (25.6) |  |
| ≥30 | 37 | (46.8) | 43 | (43.0) | 0.8 | 144 | (48.5) | 257 | (49.9) | 0.5 |
| missing |  |  |  |  |  | 1 |  | 4 |  |  |
| **OC use** |  |  |  |  |  |  |  |  |  |  |
| never | 23 | (29.1) | 31 | (31.0) |  | 88 | (29.5) | 170 | (32.8) |  |
| ever | 56 | (70.9) | 69 | (69.0) | 0.8 | 210 | (70.5) | 348 | (67.1) | 0.3 |
| missing |  |  |  |  |  |  |  | 1 |  |  |
| **Parity** |  |  |  |  |  |  |  |  |  |  |
| 0 | 18 | (22.8) | 24 | (24.0) |  | 57 | (19.1) | 101 | (19.5) |  |
| 1 | 14 | (17.7) | 15 | (15.0) |  | 36 | (12.1) | 40 | (7.7) |  |
| ≥ 2 | 47 | (59.5) | 62 | (62.0) | 0.8 | 205 | (68.8) | 378 | (72.8) | 0.1 |
| **Age at Menarche** |  |  |  |  |  |  |  |  |  |  |
| ≤ 11 | 19 | (24.1) | 23 | (23.0) |  | 71 | (24.0) | 135 | (26.2) |  |
| 12-13 | 38 | (48.1) | 51 | (51.0) |  | 143 | (48.3) | 239 | (46.4) |  |
| 14+ | 22 | (27.8) | 26 | (26.0) | 0.9 | 82 | (27.7) | 141 | (27.4) | 0.7 |
| Missing |  |  |  |  |  | 2 |  | 4 |  |  |
| **Smoking** |  |  |  |  |  |  |  |  |  |  |
| never | 50 | (63.3) | 66 | (66.0) |  | 197 | (66.1) | 342 | (66.4) |  |
| ever | 29 | (36.7) | 34 | (34.0) | 0.7 | 101 | (33.9) | 177 | (34.4) | 0.9 |
| missing |  |  |  |  |  |  |  |  |  |  |
| **^c^Family History any cancer (FDR &/or SDR)** | |  |  |  |  |  |  |  |  |  |
| no | 27 | (34.2) | 26 | (26.0) |  | 92 | (30.9) | 193 | (37.2) |  |
| yes | 52 | (65.8) | 74 | (74.0) | 0.2 | 206 | (69.1) | 326 | (62.8) | 0.07 |

^a^ Ns may not sum to the total because of missing or unknown data; proportions (%) sum to 100% of observations with data available, and excludes missing/unknowns

^b^ P-values are age-adjusted comparisons between each subset versus None; p-Trend across ordered groups (BMI, Parity, age at menarche) or logistic regression models for binary variables

^c^ Family history of cancer reported in at least one first-degree (FDR) and/or second-degree (SDR) relative

Table S3: The number and proportion of endometrial cancer cases with leiomyomas, adenomyosis and/or endometriosis

|  | **Adenomyosis & Endometriosis** | **Endometriosis/No Adenomyosis** | **Adenomyosis/No Endometriosis** | **Neither Adenomyosis nor Endometriosis** | **Total - row** |
| --- | --- | --- | --- | --- | --- |
| **Leiomyomas** | N (% of 1399) | N (% of 1399) | N (% of 1399) | N (% of 1399) | N (% of 1399) |
| *Observed Frequencies* | |  |  |  |  |
| Yes | 70 (5.0%) | 53 (3.8%) | 309 (22.1%) | 385 (27.5%) | **817** (58.4%) |
| No | 19 (1.4%) | 37 (2.6%) | 174 (12.4%) | 352 (25.2%) | **582** (41.6%) |
| ***Total - column*** | ***89 (6.4%)*** | ***90 (6.4%)*** | ***483 (34.5%)*** | ***737 (52.7%)*** | **1399** |
| *^a^Expected Frequencies* | |  |  |  |  |
| Yes | 43 (3.1%) | 62 (4.4%) | 291 (20.8%) | 421 (30.1%) | 817 (58.4%) |
| No | 30 (2.2%) | 44 (3.2%) | 208 (14.8%) | 300 (21.4%) | 582 (41.6%) |
| ***Total - column*** | ***73 (5.2%)*** | ***106 (7.6%)*** | ***499 (35.7%)*** | ***721 (51.6%)*** | ***1399*** |

^a^Expected Frequencies are based on the null hypothesis of independence of proportions using Maximum Likelihood goodness of fit distribution

Table S4: Pairwise comparisons for coexistence of conditions using all available data and self-reported where available.

Leiomyomas vs Adenomyosis (all available data)

|  | Leiomyomas | |  |  |  |
| --- | --- | --- | --- | --- | --- |
| Adenomyosis | Yes | No | Total | % Yes | ^a^P |
| Yes | 379 | 193 | **572** | 66.3 | 7.0x10^-7^ |
| No | 438 | 389 | **827** | 53.0 |  |
| ***Total*** | ***817*** | ***582*** | **1399** |  |  |

Leiomyomas vs Adenomyosis (self-reported leiomyomas only)

|  | Leiomyomas (self-reported) | | |  |  |
| --- | --- | --- | --- | --- | --- |
| Adenomyosis | Yes | No | Total | % Yes | ^a^P |
| Yes | 142 | 430 | **572** | 24.8 | 0.007 |
| No | 156 | 671 | **827** | 18.9 |  |
| ***Total*** | ***298*** | ***1101*** | **1399** |  |  |

Adenomyosis vs Endometriosis (all available data)

|  | Endometriosis | |  |  |  |
| --- | --- | --- | --- | --- | --- |
| Adenomyosis | Yes | No | Total | % Yes | ^a^P |
| Yes | 89 | 483 | **572** | 15.6 | 0.01 |
| No | 90 | 737 | **827** | 10.9 |  |
| ***Total*** | ***179*** | ***1220*** | **1399** |  |  |

Adenomyosis vs Endometriosis (self-reported endometriosis only)

|  | Endometriosis (self-reported only) | | | |  |
| --- | --- | --- | --- | --- | --- |
| Adenomyosis | Yes | No/Unk | Total | % Yes | ^a^P |
| Yes | 40 | 532 | **572** | 7.0 | 0.07 |
| No | 39 | 788 | **827** | 4.7 |  |
| ***Total*** | ***79*** | ***1320*** | **1399** |  |  |

Leiomyomas vs Endometriosis (all available data)

|  | Endometriosis | |  |  |  |
| --- | --- | --- | --- | --- | --- |
| Leiomyomas | Yes | No | Total | % Yes | ^a^P |
| Yes | 123 | 694 | **817** | 15.1 | 0.004 |
| No | 57 | 525 | **582** | 9.8 |  |
| ***Total*** | ***180*** | ***1219*** | **1399** |  |  |

Leiomyomas vs Endometriosis (self-reported only)

| Leiomyomas (self-reported only) | Endometriosis (self-reported only) | | | |  |
| --- | --- | --- | --- | --- | --- |
|  | Yes | No/Unk | Total | % Yes | ^a^P |
| Yes | 32 | 266 | **298** | 10.7 | 1.8 x 10^-5^ |
| No/Unk | 47 | 1054 | **1101** | 4.3 |  |
| ***Total*** | ***79*** | ***1320*** | **1399** |  |  |

^a^P values are based on pair-wise comparisons of proportions

Table S5: Comparison of epidemiological characteristics between EC patients with none versus any combination of leiomyomas, adenomyosis or endometriosis

| **Characteristics** | **None** |  | **Leiomyomas only** | | | **Adenomyosis only** | | | **Endometriosis only** | | | **Leiomyomas & Adenomyosis** | | | **Leiomyomas & Endometriosis** | | | **Adenomyosis & Endometriosis** | | | **All three conditions** | | |
| --- | --- | --- | --- | --- | --- | --- | --- | --- | --- | --- | --- | --- | --- | --- | --- | --- | --- | --- | --- | --- | --- | --- | --- |
|  | **^a^N** | **%** | **^a^N** | **%** | **^b^P** | **^a^N** | **%** | **^b^P** | **^a^N** | **%** | **^b^P** | **^a^N** | **%** | **^b^P** | **^a^N** | **%** | **^b^P** | **^a^N** | **%** | **^b^P** | **^a^N** | **%** | **^b^P** |
| **Subset of all cases (n=1399)** | 352 | (25.2) | 385 | (27.5) |  | 174 | (12.4) |  | 37 | (2.6) |  | 309 | (22.1) |  | 53 | (3.8) |  | 19 | (1.4) |  | 70 | (5.0) |  |
| **Mean age at EC diagnosis (range)** | 61.4 (26.4 -79.9) | | 62.6 (32.9-79.9) | | 0.08 | 61.1 (35.7-79.7) | | 0.8 | 56.5 (36.5-78.2) | | 0.008 | 62.1 (31.9-80.0) | | 0.3 | 59.1 (28.9-78.9) | | 0.1 | 54.9 (34.8-73.6) | | 0.009 | 56.2 (37.1-76.8) | | 0.0001 |
| **BMI** |  |  |  |  |  |  |  |  |  |  |  |  |  |  |  |  |  |  |  |  |  |  |  |
| <25 | 86 | (24.5) | 100 | (26.3) |  | 37 | (21.4) |  | 9 | (24.3) |  | 69 | (22.3) |  | 16 | (30.2) |  | 6 | (31.6) |  | 24 | (34.3) |  |
| 25-29.9 | 100 | (28.5) | 100 | (26.3) |  | 38 | (22.0) |  | 9 | (24.3) |  | 73 | (23.6) |  | 16 | (30.2) |  | 6 | (31.6) |  | 13 | (18.6) |  |
| ≥30 | 165 | (47.0) | 180 | (47.4) | 0.9 | 98 | (56.6) | 0.1 | 19 | (51.4) | 0.9 | 167 | (54.0) | 0.1 | 21 | (39.6) | 0.1 | 7 | (36.8) | 0.2 | 33 | (47.1) | 0.1 |
| **OC use** |  |  |  |  |  |  |  |  |  |  |  |  |  |  |  |  |  |  |  |  |  |  |  |
| never | 130 | (36.9) | 140 | (36.4) |  | 51 | (29.3) |  | 15 | (40.5) |  | 81 | (26.3) |  | 22 | (41.5) |  | 2 | (10.5) |  | 15 | (21.4) |  |
| ever | 222 | (63.1) | 245 | (63.6) | 0.7 | 123 | (70.7) | 0.09 | 22 | (59.5) | 0.6 | 227 | (73.7) | 0.002 | 31 | (58.5) | 0.4 | 17 | (89.5) | 0.04 | 55 | (78.6) | 0.02 |
| **Parity** |  |  |  |  |  |  |  |  |  |  |  |  |  |  |  |  |  |  |  |  |  |  |  |
| 0 | 64 | (18.2) | 94 | (24.4) |  | 10 | (5.7) |  | 11 | (29.7) |  | 37 | (12.0) |  | 13 | (24.5) |  | 4 | (21.1) |  | 14 | (20.0) |  |
| 1 | 38 | (10.8) | 33 | (8.6) |  | 14 | (8.0) |  | 8 | (21.6) |  | 27 | (8.7) |  | 7 | (13.2) |  | 4 | (21.1) |  | 9 | (12.9) |  |
| ≥ 2 | 250 | (71.0) | 258 | (67.0) | 0.03 | 150 | (86.2) | 2.24E-05 | 18 | (48.6) | 0.1 | 245 | (79.3) | 0.02 | 33 | (62.3) | 0.4 | 11 | (57.9) | 0.9 | 47 | (67.1) | 0.5 |
| **Age at Menarche** |  |  |  |  |  |  |  |  |  |  |  |  |  |  |  |  |  |  |  |  |  |  |  |
| ≤ 11 | 68 | (19.5) | 92 | (24.0) |  | 24 | (14.0) |  | 10 | (27.0) |  | 88 | (28.9) |  | 10 | (18.9) |  | 6 | (31.6) |  | 16 | (22.9) |  |
| 12-13 | 167 | (48.0) | 183 | (47.8) |  | 92 | (53.8) |  | 19 | (51.4) |  | 135 | (44.3) |  | 29 | (54.7) |  | 6 | (31.6) |  | 35 | (50.0) |  |
| 14+ | 113 | (32.5) | 108 | (28.2) | 0.06 | 55 | (32.2) | 0.4 | 8 | (21.6) | 0.3 | 82 | (26.9) | 0.007 | 14 | (26.4) | 0.7 | 7 | (36.8) | 0.9 | 19 | (27.1) | 0.7 |
| **Smoking** |  |  |  |  |  |  |  |  |  |  |  |  |  |  |  |  |  |  |  |  |  |  |  |
| never | 220 | (62.7) | 249 | (64.7) |  | 116 | (66.7) |  | 21 | (56.8) |  | 207 | (67.0) |  | 33 | (62.3) |  | 12 | (63.2) |  | 50 | (71.4) |  |
| ever | 131 | (37.3) | 136 | (35.3) | 0.7 | 58 | (33.3) | 0.3 | 16 | (43.2) | 0.7 | 102 | (33.0) | 0.3 | 20 | (37.7) | 0.9 | 7 | (36.8) | 0.8 | 20 | (28.6) | 0.09 |
| **^c^Family History any cancer (FDR &/or SDR)** | | | |  |  |  |  |  |  |  |  |  |  |  |  |  |  |  |  |  |  |  |  |
| no | 139 | (39.5) | 135 | (35.1) |  | 62 | (35.6) |  | 6 | (16.2) |  | 109 | (35.3) |  | 15 | (28.3) |  | 6 | (31.6) |  | 26 | (37.1) |  |
| yes | 213 | (60.5) | 250 | (64.9) | 0.2 | 112 | (64.4) | 0.4 | 31 | (83.8) | 0.01 | 200 | (64.7) | 0.3 | 38 | (71.7) | 0.1 | 13 | (68.4) | 0.5 | 44 | (62.9) | 0.7 |

^a^ Ns may not sum to the total because of missing or unknown data; proportions (%) sum to 100% of observations where data available and excludes missing/unknowns

^b^ P-values are age-adjusted comparisons between each subset versus None; p-Trend across ordered groups (BMI, Parity, age at menarche) or logistic regression models for binary variables

^c^ Family history of cancer reported in at least one first- or second-degree relative

Table S6: Characteristics of ovarian tumors and gynecologic conditions among EC patients with prior/concurrent ovarian cancer

| Patient # | OC-Prior/Concurrent | Proband age at cancer diagnosis (EC ; OC) | EC Histological Subtype/Grade | ^a^OC Histological Subtype/Grade | ^b^Lymphovascular Space Invasion | ^c^Endometriosis | ^d^Endometriosis location | ^c^Adenomyosis | ^c^Leiomyoma |
| --- | --- | --- | --- | --- | --- | --- | --- | --- | --- |
| 1 | Concurrent | 53 | Endometrioid, Grade 1 | Mixed - Clear Cell, Mucinous & Endometrioid, grade not recorded | no | yes | ovary | unk | unk |
| 2 | Concurrent | 46 | Endometrioid, Grade 1 | Endometrioid, Grade 2 | no | unk | na | yes | yes |
| 3 | Concurrent | 37 | Endometrioid, Grade 2 | Endometrioid, Grade 1 | yes | yes | ovary | yes | unk |
| 4 | Concurrent | 54 | Endometrioid, Grade 1 | Endometrioid, Grade 1 | unk | yes | ovary | yes | yes |
| 5 | Concurrent | 45 | Mixed - Endometrioid, with some Clear Cell differentiation, Grade 3 | Endometrioid, Grade 1 | yes | yes | uterine serosa | unk | unk |
| 6 | Concurrent | 72 | Endometrioid, Grade 2 | Endometrioid, Grade 3 | no | unk | na | unk | yes |
| 7 | Concurrent | 53 | Carcinosarcoma (MMMT) | Mixed - Endometrioid & Serous, Grade 2 | no | unk | na | unk | yes |
| 8 | Prior | 33 ; 29 | Endometrioid, Grade 1 | Endometrioid, Grade not recorded | no | unk | na | unk | unk |
| 9 | Concurrent | 38 | Endometrioid, Grade 1 | Endometrioid, Grade 2 | yes | yes | bladder | unk | unk |
| 10 | Concurrent | 58 | Endometrioid, Grade 2 | Mixed - Endometrioid & Clear Cell, Grade 3 | no | unk | na | unk | yes |
| 11 | Concurrent | 76 | Endometrioid, Grade 1 | Endometrioid, Grade 1 | no | unk | na | unk | yes |
| 12 | Concurrent | 66 | Endometrioid, Grade 1 | Endometrioid, Grade 1 | yes | yes | ovary | yes | yes |
| 13 | Concurrent | 64 | Serous (>5%) | Mixed - Serous (70%) & clear cell (25%) | yes | unk | na | unk | yes |
| 14 | Concurrent | 45 | Endometrioid, Grade 1 | Mixed - Serous (60-70%) and Endometrioid (30-40%), Grade 2 | no | unk | na | unk | yes |
| 15 | Concurrent | 60 | Clear Cell (>10%), no serous | Mixed - Endometrioid & Clear Cell, Grade 3 | yes | unk | na | unk | yes |
| 16 | Concurrent | 75 | Endometrioid, Grade 2 | Adenocarcinoma, Grade 2 | yes | unk | na | yes | unk |
| 17 | Concurrent | 37 | Endometrioid, Grade 1 | Endometrioid with Squamous differentiation, Grade 1 | no | no | na | yes | unk |
| 18 | Concurrent | 47 | Endometrioid, Grade 2 | Endometrioid, Grade 3 | no | yes | ovary | unk | yes |
| 19 | Concurrent | 46 | Endometrioid, Grade 2 | Endometrioid with serous features, Grade 2 | no | no | na | yes | unk |
| 20 | Concurrent | 55 | Endometrioid, Grade 1 | Endometrioid with Squamous differentiation, Grade 2 | no | yes | ovary | yes | unk |
| 21 | Concurrent | 67 | Carcinosarcoma (MMMT) | Mixed - Clear Cell and Endometrioid with serous papillary foci | unk | yes | ovary | yes | yes |
| 22 | Concurrent | 55 | Endometrioid, Grade 2 | Mixed - Mucinous & Endometrioid, Grade 2 | no | yes | ovary | unk | unk |
| 23 | Concurrent | 48 | Endometrioid, Grade 1 | Endometrioid, Grade 1 | unk | unk | na | unk | unk |
| 24 | Prior | 59 ; 57 | Carcinosarcoma (MMMT) | Granulosa cell tumor, Grade not recorded | no | yes | na | yes | yes |
| 25 | Concurrent | 60 | Endometrioid, Grade 3 | Endometrioid, Grade 2 | yes | yes | ovary | unk | unk |
| 26 | Concurrent | 72 | Endometrioid, Grade 2 | Endometrioid, Grade 2 | yes | unk | na | unk | yes |
| 27 | Concurrent | 49 | Endometrioid, Grade 2 | Clear Cell, Grade 3 | no | unk | na | unk | unk |
| 28 | Concurrent | 58 | Endometrioid, Grade 1 | Mucinous, Grade 2 | yes | unk | na | unk | yes |
| 29 | Concurrent | 50 | Endometrioid, Grade 2 | Endometrioid, Grade 2 | no | unk | na | unk | no |
| 30 | Concurrent | 54 | Endometrioid, Grade 1 | Endometrioid with Squamous differentiation, Grade 1 | no | yes | ovary | yes | unk |

^a^ Ovarian cancer histology was reported in EC pathology reports where biopsies/tumor samples were obtained for synchronous EC and ovarian cancers. For prior ovarian cancers, EC pathology reports had limited information on tumor histology, or obtained from clinical notes in EC pathology reports.

^b^ Lymphovascular space invasion of endometrial tumors were obtained from EC pathology reports; yes indicates LVSI, no indicates no evidence of LVSI; unk indicates no mention of LVSI in pathology reports

^c^ Evidence of endometriosis, adenomyosis and leiomyoma data were obtained from EC pathology reports; yes indicates evidence of gynecologic condition reported; no indicates absence of condition specifically reported by pathologist; unk indicates no mention of gynecologic condition in pathology reports

^d^ Location of Endometriosis was obtained from EC pathology reports where specifically stated; na indicates location not specified in pathology report, or endometriosis not indicated in pathology report
